# Ebanga™: The most recent FDA-approved drug for treating Ebola

**DOI:** 10.3389/fphar.2023.1083429

**Published:** 2023-03-08

**Authors:** Elahe Taki, Roya Ghanavati, Tahereh Navidifar, Shirin Dashtbin, Mohsen Heidary, Marjan Moghadamnia

**Affiliations:** ^1^ Department of Microbiology, School of Medicine, Tehran University of Medical Sciences, Tehran, Iran; ^2^ Behbahan Faculty of Medical Sciences, Behbahan, Iran; ^3^ Department of Basic Sciences, Shoushtar Faculty of Medical Sciences, Shoushtar, Iran; ^4^ Department of Microbiology, School of Medicine, Iran University of Medical Sciences, Tehran, Iran; ^5^ Microbial Biotechnology Research Center, Iran University of Medical Sciences, Tehran, Iran; ^6^ Department of Laboratory Sciences, School of Paramedical Sciences, Sabzevar University of Medical Sciences, Sabzevar, Iran; ^7^ Leishmaniasis Research Center, Sabzevar University of Medical Sciences, Sabzevar, Iran; ^8^ Department of Clinical Pharmacy, School of Pharmacy, Tehran University of Medical Sciences, Tehran, Iran

**Keywords:** Ebola virus, treatment, monoclonal antibody, ansuvimab-zykl, mAb114, Ebanga^TM^

## Abstract

*Ebolavirus* (EBOV) is a virulent pathogen that causes Ebola virus disease (EVD), which is a life-threatening human condition with a fatality rate of up to 90%. Since the first outbreak in Africa in 1976, several outbreaks and epidemics of EBOV have occurred across the globe. While EVD is recognized as a serious threat to human health and outbreaks occur almost every year, the treatment options for the disease are limited. In designing therapeutic strategies against EBOV infection, viral structural proteins, such as glycoprotein (GP), could be an excellent target for neutralizing the virus. According to the latest research, GP-specific antibodies are the most efficient post-exposure treatments for EVD. Ansuvimab-zykl, i.e., mAb114 (Ebanga™), is a recent FDA-approved human immunoglobulin monoclonal antibody targeting EBOV GP. This review provides a brief overview of the pharmacological effects and safety profile of ansuvimab in clinical trials and provides insights into the precise mechanism of this new drug for treating EVD.

## 1 Introduction

*Ebolavirus* (EBOV) is a virulent pathogen causing Ebola virus disease (EVD). The disease was first discovered in 1976 in a village close to the Ebola River in the Democratic Republic of the Congo. EVD is a hemorrhagic fever virus infection that has induced several outbreaks, primarily in Africa. The advanced stage of the infection is initially characterized by a variety of symptoms including coughing and chest pain (in the respiratory system), diarrhea, abdominal pain, vomiting (in the gastrointestinal tract), and confusion and headache (in the nervous system). In severe cases of infection, multi-organ failure occurs (Paessler and Walker, 2013; World Health Organization, 2020). *Ebolavirus* and *Marburgvirus*, which belong to the order *Mononegavirales* and the family *Filoviridae*, are responsible for hemorrhagic fever and result in a high rate of mortality. Due to the high burden of mortality, transmissibility, and potential aerosol infectivity, EBOV is classified as a biosafety level 4 agent (Di Paola et al., 2020). Based on substantial antigenicity variations and genetic information, the genus *Ebolavirus* is subdivided into six species: *Sudan virus* (SUDV), *Zaire ebolavirus* (EBOV), *Bundibugyo virus* (BDBV), *Tai Forest virus* (TAFV), and *Reston virus* (RESTV). SUDV and EBOV have been reported as the predominant species and are associated with higher pathogenicity, outbreaks, and mortality (up to 90%) compared to other species of EBOVs (Kadanali and Karagoz, 2015). BDBV and TAFV, like EBOV, mostly infect humans while RESTV mainly affects pigs. *Bombali virus* (BOMV), the most recent EBOV, has been detected in bat samples in Sierra Leone (Goldstein et al., 2018). Virtually, all human cases of EVD are related to the emergence or recurrence of the SUDV and EBOV in the regions of Gabon, the Republic of the Congo, Sudan, and Uganda (World Health Organization, 2020).

Currently, no exact origin has been identified for EBOV, but it is likely that animals infected through direct contact with the suspected vector host or other animals (e.g., chimpanzees, monkeys, and apes) and even humans are the main cause of EBOV circulation (Centers for Disease Control and Prevention, 2014). The close contact of individuals with infected animals can disseminate EBOV among the human population (Malvy et al., 2019). African fruit bats (*Epomops franqueti*, *Hypsignathus monstrosus*, *Rousettus aegyptiacus*, and *Myonycteris torquata*) are presumably involved in the spread of EBOV as a vector or even as a reservoir host (Feldmann and Geisbert, 2011). The secondary transmission of the virus from human to human occurs by direct contact with body fluids, such as blood, saliva, semen, and breast milk, of patients during EBOV epidemics (Chughtai et al., 2016).

Since the first EBOV outbreak was recognized in 1976, 11 outbreaks have been reported, particularly in the central regions and recently in the west regions of Africa. The largest EBOV outbreak in history that became a global epidemic within months happened during 2014–2016 in West Africa, and up to 28,000 cases of EVD and 11,000 deaths were recorded in Guinea, Liberia, and Sierra Leone (Sivanandy et al., 2022). The recent EBOV outbreak in Guinea in 2021 and the continuing epidemic in Mbandaka and the Equateur Provinces of the Democratic Republic of the Congo since 2022, which are connected to the 2018–2020 outbreak, emphasize the necessity for ongoing attention and continuous surveillance (World Health Organization, 2022).

Despite the high annual frequency of unpredictable EBOV outbreaks, limited effective treatment options are available. A number of therapeutic molecules, including small interfering RNAs, ion channel inhibitors, small-molecule inhibitors, antibodies, and interferons, have been evaluated through different *in vitro* studies or clinical trials to understand the efficacy of drugs against EBOV (Chakraborty, 2021). The most investigated antiviral target for EBOV that could serve as a therapeutic option is the entrance of the virus through glycoprotein (GP) subunits. EBOV GP can be inhibited by neutralizing antibodies, synthetic chemicals, and organic substances. Existing research indicates that GP-specific antibodies are the most successful post-exposure treatments. The current monoclonal antibody (mAb)-based treatments that were developed as GP inhibitors include three drugs: ZMapp, REGN-EB3 (Inmazeb™), and ansuvimab-zykl or mAb114 (Ebanga™) (Sivanandy et al., 2022). ZMapp, which is composed of three different mAbs (4G7-13C6-2G4), targets the surface GP of the virion and inhibits the progression of EVD (Authors Anonymous, 2019). REGN-EB3, the first FDA-approved drug for adults and children, consists of three full human mAbs (REGN3470-REGN3471-REGN3479) that block the attachment of the virus to host cell proteins (US Food and Drug Administration, 2022). Ansuvimab-zykl (ansuvimab), which was formerly named mAb114, was developed by the Vaccine Research Center with the support of the US National Institute of Health for the treatment of EVD and is produced by Ridgeback Biotherapeutics in the United States of America under the name Ebanga™. The safety and efficacy of ansuvimab was evaluated during the Pamoja Tulinde Maisha (PALM) phase II/III study, and following the successful results, the drug was approved by the United States Food and Drug Administration (USFDA) on 21 December 2020 for the treatment of EVD (Mulangu et al., 2019; FDA, 2020). Ansuvimab is a human immunoglobulin mAb obtained from memory B cells of the survivors of the Kikwit EVD epidemic and is specifically used to treat the *Zaire* EBOV (Levine, 2019); its activation is dependent on a low intracellular pH environment. Ansuvimab targets the conserved region of amino acids on receptor-binding domains (RBDs) of GP and prevents the binding of virions to the late endosomal Niemann–Pick intracellular cholesterol transporter-1 (NPC-1) protein, which is the receptor for GP in host cells (Carette et al., 2011). Due to the vital role of RBD in the infectivity of EBOV, ansuvimab binding to this domain could mitigate the risk of escape mutants as alterations in RBD can result in a decline in viral fitness (Gaudinski et al., 2019). The present review elucidates various aspects of ansuvimab, including its mechanism of action against EBOV, clinical implications, efficacy, and resistance, in patients who have used this drug.

## 2 Structure and antiviral properties of ansuvimab

### 2.1 Development and the antiviral attributes of ansuvimab

Ansuvimab was developed by Ridgeback Biotherapeutics to treat EBOV infections in adults and pediatric patients (Authors Anonymous, 2021). In December 2018, the company entered into a patent license agreement with the US National Institute of Allergy and Infectious Diseases to use ansuvimab in the treatment of EVD. Between September 2019 and April 2020, the US Department of Health and Human Services granted Ridgeback Biotherapeutics permission to manufacture ansuvimab (Lee, 2021). On 21 December 2020, after promising outcomes of the phase II/III clinical trials, ansuvimab received FDA approval (FDA, 2020; World Health Organization, 2020).

Ansuvimab is a single mAb initially isolated from memory B cells of two patients who survived the EBOV outbreak in Kikwit in 1995 and maintained antibodies against the EBOV surface GP for 11 years after the infection (Levine, 2019). The enzyme-linked immunosorbent assay (ELISA) and cell-based assay on human embryonic kidney 293T (HEK293T) cells showed that the sera of these survivors had potent virus-binding and -neutralizing activity compared with the control sera. Therefore, to investigate which antibodies induced this protection against EBOV, the memory B cells from the peripheral blood of survivors were immortalized by the Epstein–Barr virus and cultured. Finally, their mAb protection ability against the GP of EBOV was examined. Among the 40 memory B cell clones that expressed antibodies against GP *in vitro*, two clones (mAb100 and mAb114) had markedly neutralizing activity against EBOV in subsequent tests (Traggiai et al., 2004; Corti et al., 2016). The results of the plaque-reduction assay demonstrated that isolated mAbs could neutralize recent and earlier outbreak variants of EBOV and cause antibody-dependent cell-mediated cytotoxicity (ADCC) *in vitro* (Corti et al., 2016). The binding affinity of the aforementioned mAbs, compared to other mAbs, indicated that the maximum binding of mAb114 to GP was 25% higher than 13C6 (a component of the ZMapp cocktail) and approximately 50% higher than KZ52 (a prototypical human mAb specific for GP of EBOV) (Wilson et al., 2000; Qiu et al., 2014). After confirming the effectiveness of mA114, its name was changed to ansuvimab and its recombinant form was obtained by cloning the variable domains of EBOV-specific B cell receptors into a human IgG1 backbone (Krishnan et al., 2012). Ansuvimab is classified as an IgG1 subclass antibody with variable heavy chain (V3-13*01) and light chain (VK1-27*01) domains and a heavy chain complementarity-determining region 3 with a length of 13 (Corti et al., 2016).

### 2.2 Ansuvimab target pathway and action mechanism

**FIGURE 1:**
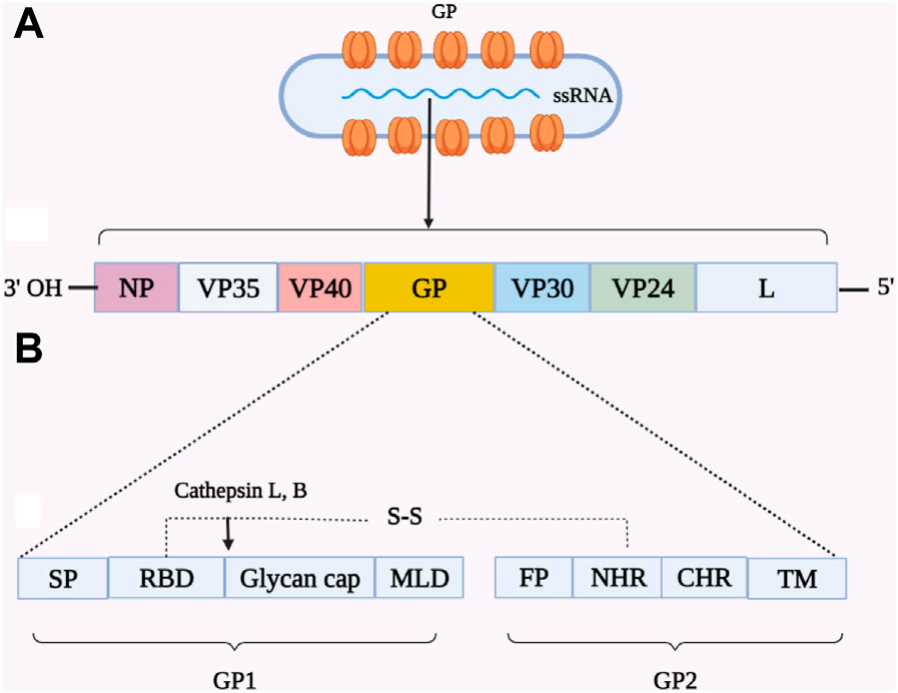
Schematic representation of the genome organization of *Ebolavirus* (EBOV) **(A)** and a linear view of the glycoprotein (GP) **(B)**. The single-stranded RNA (ssRNA) genome of EBOV is comprised of certain encoding protein genes. The viral proteins (VP), including NP (nucleoprotein) and VP24 (matrix protein), form the ribonucleoprotein complex. Other VP, such as VP40 (matrix protein), VP30 (transcriptional factor), VP35 (polymerase cofactor), and L (RNA polymerase), are involved in the structure of the virus or replication. The surface spikes on the virion are composed of a trimeric transmembrane GP that is comprised of GP1 and GP2 linked by a disulfide bond. The GP1 subunit consists of a signal peptide (SP), a receptor-binding domain (RBD), a glycan cap, and a mucin-like domain (MLD). The GP2 subunit contains a transmembrane domain (TM) anchor to two heptad repeats, namely, N-terminal (NHR) and C-terminal (CHR) heptad repeats, and a membrane-proximal external region (not shown).

EBOV contains a negative single-stranded RNA genome that encodes several structural proteins that comprise a trimeric transmembrane GP. This protein is significant because it contains putative linear, conformation-dependent, and quaternary antibody-binding epitopes (Lee et al., 2008; Misasi and Sullivan, 2021). The protomers of GP consist of GP1/GP2 heterodimers that are linked by disulfide bonds and form a chalice-shaped trimer (Figure 1). GP1 is chiefly engaged in viral attachment to the host cell receptors, while GP2 is responsible for membrane fusion. The GP1 subunit contains RBD and a “glycan cap,” which are protected by an extensively glycosylated mucin-like domain (MLD) (Misasi et al., 2016; Ghosh et al., 2021). The GP2 subunit consists of two heptad repeats (HR1 and HR2), a hydrophobic internal fusion loop (IFL), a membrane-proximal external region, a CX6CC disulfide bond motif, and a transmembrane domain. The IFL proceeds *via* the fusion of EBOV with target cell membranes. EBOV-infected cells produce secreted GP (sGP), which has 295 N-terminal amino acids but is devoid of GP2 and MLD (Wang et al., 2021).

**FIGURE 2:**
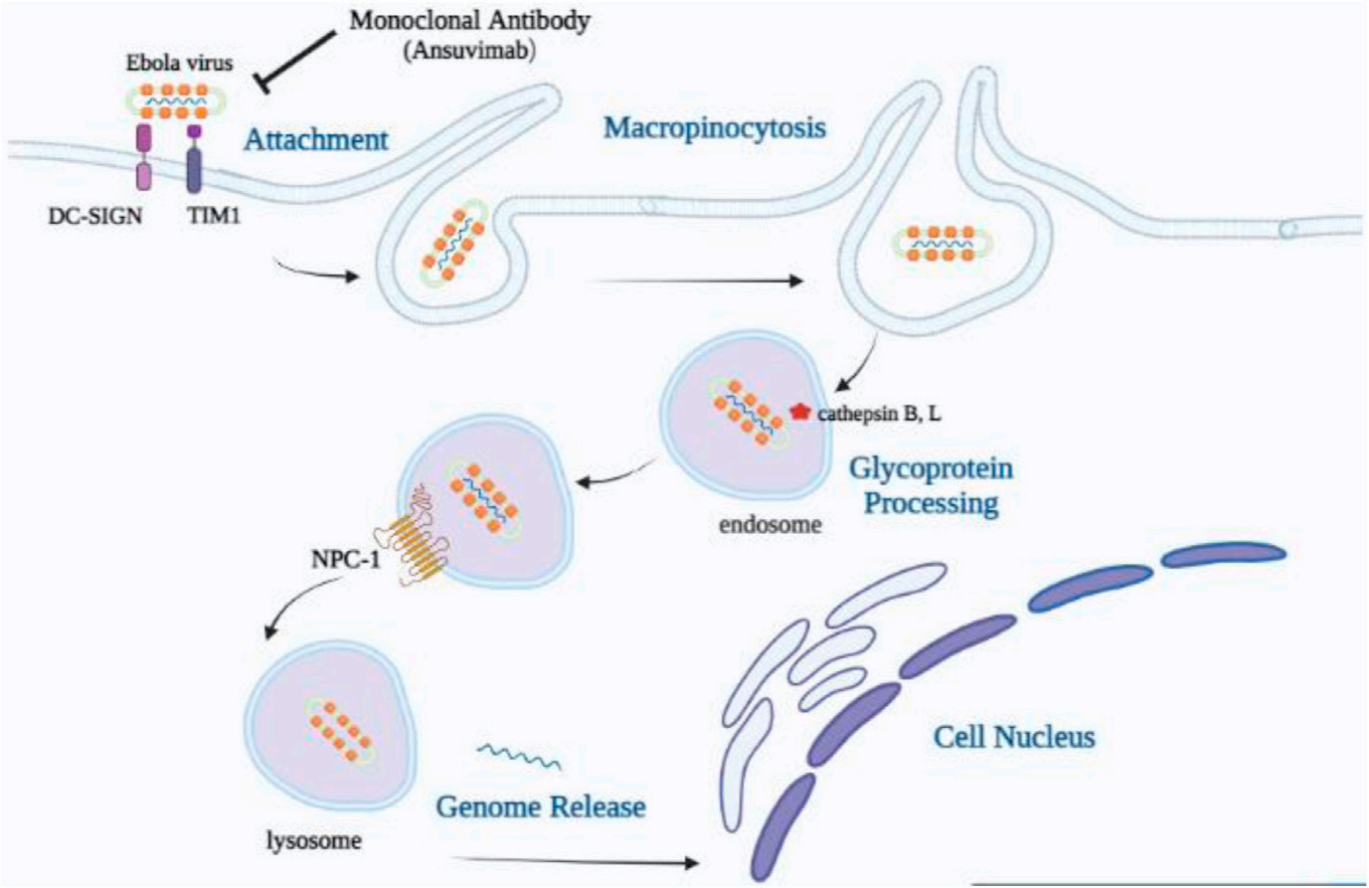
Mode of cellular uptake and entrance of the Ebola virus. Viral particles join with surface elements, T-cell immunoglobulin mucin (TIM) proteins, and dendritic cell-specific intercellular adhesion molecule-3-grabbing nonintegrin-1 (DC-SIGN) and stimulate uptake through macropinocytosis, which contributes to the trafficking to endosomes. The receptor-binding domain in the GP1 core becomes visible in the low-pH endosome by cathepsin B, L’s removal of the mucin-like domain (MLD), and the glycan cap. Viral particles with exposed RBDs can engage the host cell receptor, Niemann–Pick intracellular cholesterol transporter-1 (NPC-1) protein, which results in the release of the viral genome into the cell cytoplasm and nucleus.

EBOV attachment to cell surface molecules, including T-cell immunoglobulin mucin (TIM) proteins, C-type lectins, dendritic cell-specific intercellular adhesion molecule-3-grabbing nonintegrin-1 (DC-SIGN), and Tyro3, Axl, and MerTK (TAM) family receptor tyrosine kinases, results in virion entry into the cell by macropinocytosis (Carette et al., 2011; Lee, 2021). Next, the host endolysosome cleaves the glycan cap on GP1 and then exposes the RBD to NPC-1 through the host cysteine proteases cathepsins B and L by binding to the LEIKKPDGS (GP residues 111–119), which is located in the RBD of the GP1 subunit (Figure 2). This interaction mediates membrane fusion with the GP2 subunit and subsequently causes the release of the viral ribonucleoprotein complex into the cytoplasm (Misasi et al., 2016).

**FIGURE 3:**
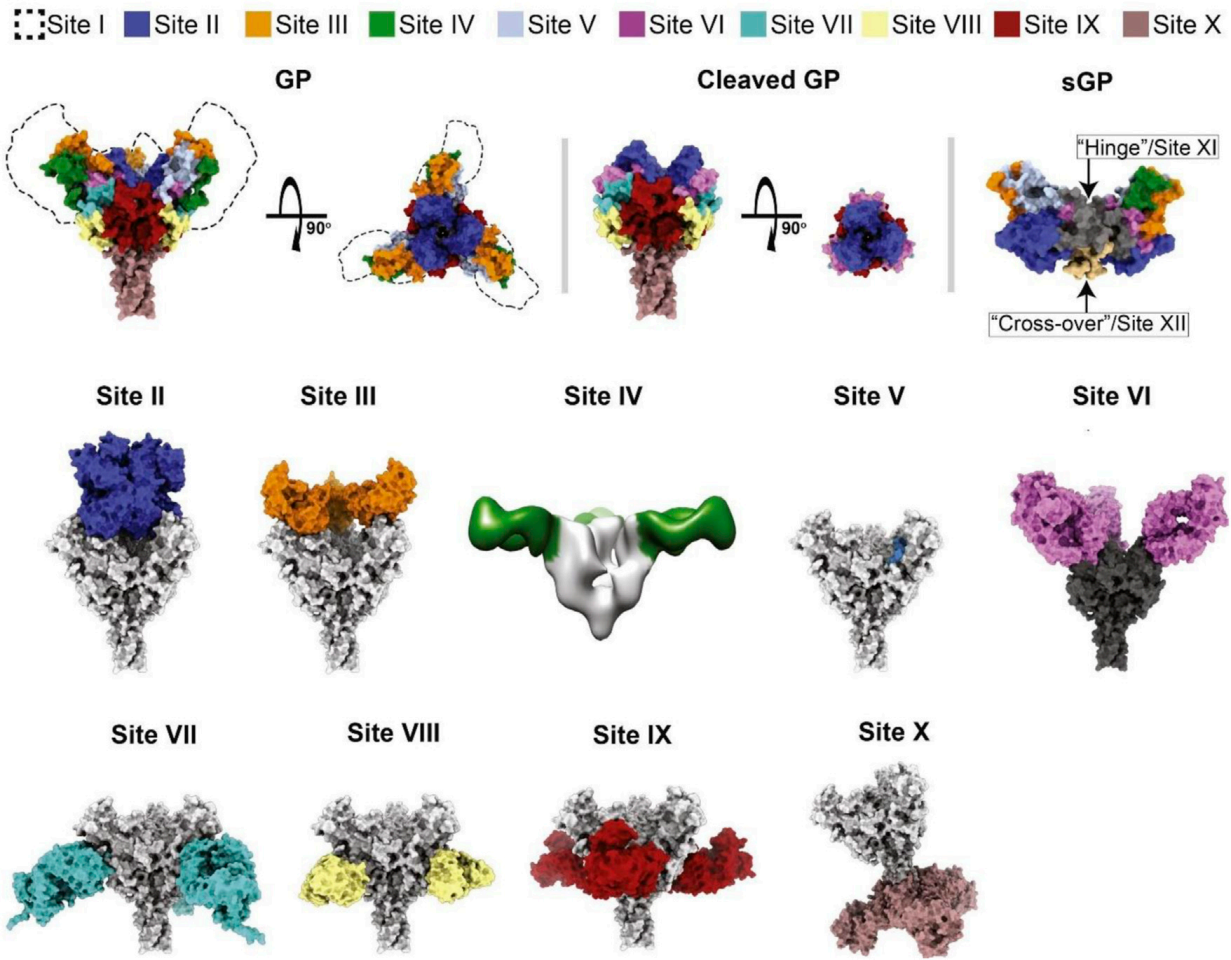
Classification scheme of the crystal structure of GP and secreted GP (sGP) of *Zaire* EBOV that defines the antigenic sites of vulnerability. The approximate location of site I and the MLD are indicated by dashed lines, while other sites are shown by specified colors (Misasi and Sullivan, 2021).

Misasi and Sullivan (2021) proposed a new antigenic site-based schema for EBOV species (*Zaire*, SUDV, and BDVB), which is based on structural motifs and known antigenic sites in GP and secreted GP. The novel schema permits antigenic variations within domains or functional aspects targeted and blocked by antibodies. These antigenic sites of vulnerability on EBOV GP entail site I (MLD), site II (GP1 core [chalice]), site III (glycan cap [top]), sites IV and V (glycan cap), site VI (RBD), site VII–IX (base of GP), and site X (HR2) (Figure 3).

Several mAbs have been determined to neutralize different sites of GP, and some of them have shown promising results in the treatment of EVD. Although the major mechanisms of virus neutralization identified for antibodies targeting the IV, V, and X sites are unknown, sites II and VI on GP could be neutralized by ansuvimab and MR78 antibodies, respectively. Site VII, which is adjacent to the cathepsin-cleavage loop, could be targeted by antibodies mimicking the β17–β18 loop of the glycan cap. Antibodies targeting site VIII primarily contact IFL and HR1A on the surface site and inhibit structural rearrangements of GP2, which are required for the fusion of the virion. While site IX antibodies are able to target the fusion loop and cathepsin-cleavage loop of GP, antibodies that make contact with sites I and III showed no neutralizing activity (Misasi and Sullivan, 2021). Since RBD plays a crucial role in viral entrance, this neutralization mechanism is an essential property of antibodies targeting site II. However, antibodies of site II must contact GP from the top to reach the recessed center of the chalice region. This limited contact could be a justification for the small number of antibodies identified in recognizing site II (Misasi and Sullivan, 2021). The antigen-binding fragments (Fab) of ansuvimab shown in the crystal structure bind within the GP chalice, perpendicular to the EBOV membrane, and make a connection not only with the glycan cap but also with the GP1 core. An evaluation of the binding modes of each Fab to GP ectodomains lacking the MLD using negative-stain electron microscopy and single-particle analysis revealed that at least two Fabs are bound to each GP trimer (Misasi et al., 2016; Cagigi et al., 2018).

Corti et al. (2016) used flow cytometry to evaluate the ADCC activity of ansuvimab in the presence of antibodies with effector cells added at a ratio of effector-to-target cell of 1:50 in EBOV GP-transduced and non-transduced HEK293T target cells. They reported ansuvimab-mediated ADCC with maximal activity at an mAb concentration of 0.03 μg/mL. One strategy to neutralize EBOV using ansuvimab is Fc-mediated binding to the virion, which facilitates some of the important immune pathways including ADCC, complement-dependent cytotoxicity (CDC), antibody-dependent cellular phagocytosis (ADCP), immune complex-mediated enhancement of antigen presentation, opsonization, and enhancement of T-cell functions (Misasi and Sullivan, 2021).

## 3 Possible mechanism of resistance to ansuvimab

Since the genome of EBOV can change over time, the development of resistance or changes in the virulence factors could lessen the clinical effectiveness of antiviral medications (Ghosh et al., 2021). Considering that the target domain of ansuvimab (RBD) in GP plays a vital role in virus infectivity, mutation in this target seems to lead to a decrease in survival of the virus (Gaudinski et al., 2019). Hence, an important matter in the possibility of developing resistance to ansuvimab is related to its binding mechanism to GP; however, there have been no investigations on ansuvimab resistance in either non-clinical or clinical settings. When administering ansuvimab, it is important to consider data on the drug susceptibility patterns of circulating EBOV strains. Moreover, patients who either do not react to treatment or experience a relapse of EVD after an initial phase of responsiveness should be aware of the risk of ansuvimab resistance (Ebanga, 2020).

## 4 Potential synergism of ansuvimab with REGN-EB3 (Inmazeb™)

Determining the synergistic effects of combining two mAbs for the treatment of EVD is challenging, and this combined approach has not been investigated in a clinical or laboratory trial (Crozier et al., 2022). Therefore, it is crucial to investigate the non-interference of the proposed products (ansuvimab and REGN-EB3) with important safety signals (Finch et al., 2021). There are significant differences between REGN-EB3 and ansuvimab in the production strategy and the mechanism of action. REGN-EB3 consists of three mAbs, namely, atoltivimab, maftivimab, and odesivimab that were acquired by immunizing VelocImmune mice, and produces fully human antibodies by encoding variable gene segments (Levine, 2019). In terms of the mechanism of action, the three aforementioned mAbs in REGN-EB3 bind simultaneously to distinct, non-overlapping epitopes and do not compete for binding to the *Zaire* EBOV GP, which interestingly decreases the occurrence of escape mutants and increases the efficacy of REGN-EB3 in virus neutralization (Sivanandy et al., 2022). As ansuvimab binds to distinct, non-overlapping epitopes in EBOV GP, REGN-EB3 is not a target and it is likely that the combination of these two drugs may have synergistic effects.

## 5 Potential interaction of ansuvimab with vaccines

Interaction between vaccines used to prevent EBOV and ansuvimab is unknown because vaccine–therapeutic interaction studies have not been conducted in humans; thus, this interaction needs to be evaluated in future investigations. Due to the potential of ansuvimab to inhibit the replication of live virus vaccines, simultaneous administration of live vaccines with ansuvimab for the prevention of EBOV infection reduces the efficacy of the vaccine (Ebanga, 2020). It has been shown that the therapeutic efficacy of the live adenovirus type 7 vaccine and new anthrax vaccine (AV7909) can be declined when used in combination with ansuvimab (DRUGBANK, 2021). Hence, to minimize such interference effects and according to current vaccination recommendations, an interval between the administration of ansuvimab and a live vaccination is essential (Ebanga, 2020). According to results obtained from the PALM trial, the efficacy of ansuvimab among subjects receiving a recombinant live vaccine prior to their enrollment was similar to that of patients who did not receive any vaccinations (Mulangu et al., 2019). Additionally, the concomitant administration of ansuvimab with some drugs, such as abciximab, adalimumab, aducanumab, alemtuzumab, amivantamab, and anifrolumab, could increase the risk or severity of adverse effects. Moreover, the combination of the anthrax vaccine with ansuvimab decreased its therapeutic effectiveness (Ridgeback Biotherapeutics, 2021; Mulangu et al., 2022).

## 6 Pharmacodynamics and pharmacokinetics

Ansuvimab inhibits binding between the EBOV GP and NPC-1 receptor by targeting the LEIKKPDGS epitope in the RBD of the GP1 subunit and thereby facilitates membrane fusion in viral entry (Misasi et al., 2016; Ridgeback Biotherapeutics, 2021). Cryo-electron microscopy showed that ansuvimab binds to the GP core and glycan cap domains in a position almost perpendicular to the viral membrane. Biolayer interferometry also revealed that at pH 7.4 (dissociation constant [K_D_] of 0.2 nM) and pH 5.3 (K_D_ of 0.6 nM), ansuvimab has a high affinity for GP1 devoid of the mucin domain (Misasi et al., 2016). Ansuvimab blocked GP1 binding to NPC-1 at a half maximal inhibitory concentration (IC_50_) of 0.09 μg/mL and thus inhibited EBOV entry into the host cell (Ridgeback Biotherapeutics, 2021). Results of a plaque-reduction neutralization assay with *Zaire* EBOV Mayinga represented half maximal effective concentration (EC_50_) values of 0.06 μg/mL for ansuvimab. However, in a lentivirus infectivity assay with *Zaire* EBOV Mayinga and *Zaire* EBOV Makona, the EC_50_ values were 0.09 and 0.15 μg/mL, respectively (Ridgeback Biotherapeutics, 2021). The results of flow cytometry demonstrated a maximal ADCC activity of ansuvimab at a concentration of 0.03 μg/mL against GP-transfected target cells (Corti et al., 2016). Administration of 50 mg/kg of ansuvimab to an animal model (rhesus macaques) on days 1–3 resulted in the survival of all animals (n = 3) following exposure to a lethal dose of EBOV. Moreover, ansuvimab protected rhesus macaques from death when given as late as 5 days after exposure to a lethal dose of EBOV (Corti et al., 2016).

The pharmacodynamic response–time course and exposure–response relationship of ansuvimab are unknown. In a clinical trial involving 18 healthy individuals, the pharmacokinetic profile of this mAb, i.e., ansuvimab, was comparable to other IgG1 mAbs (Lee, 2021; Ridgeback Biotherapeutics, 2021). Furthermore, the pharmacokinetic properties of ansuvimab were investigated by intravenous (IV) administration of ansuvimab at 5, 25, and 50 mg/kg concentrations in three, five, and five cases, respectively, in healthy volunteers during a phase I pharmacokinetic trial (VRC 608). The pharmacokinetic properties of ansuvimab, including maximum concentration (Cmax), time of maximum concentration (Tmax), area under the curve on days 0–28 (AUC_0-28d_)_,_ β half-life (t_1/2β_), and mean serum concentrations on days 0–28, were measured in each of the three concentrations mentioned previously. In healthy volunteers receiving 50 mg/kg of ansuvimab, the Cmax and Tmax were 1961.21 μg/mL and 2.75 h, respectively, AUC_0-28d_ was calculated as 185,88 μg day/mL, and t_1/2β_ was reported as 23.6 days. However, the average half-life of ansuvimab at all doses was 24.2 days. With a daily clearance rate of 115 mL, the average concentration in the 50 mg/kg group for the first 28 days was 664 g/mL. The result of the phase I dose-escalation study showed that at a dosage up to 50 mg/kg, all ansuvimab levels were well tolerated and there was no sign of infusion responses. The pharmacokinetic profile of ansuvimab also indicated dose-dependent linearity and low inter-participant variation of ansuvimab within a single dosage (Gaudinski et al., 2019). However, according to clinical evidence, the patient’s demographic features and comorbidities such as age, kidney disease, and hepatic impairment did not affect the pharmacokinetics of this mAb (Ridgeback Biotherapeutics, 2021).

## 7 Description and clinical considerations in specific populations

Preparation and administration of ansuvimab must be conducted under the supervision of healthcare professionals. Ansuvimab is marketed as an off-white to white lyophilized powder and needs reconstitution and dilution for IV administration. The effective dose for both adults and pediatric patients is 50 mg/kg administered within 1 hour. Each single-dose vial of this mAb contains ansuvimab (400 mg), L-histidine (12.4 mg), L-histidine HCl (16.8 mg), polysorbate 80 (1.6 mg), and sucrose (657 mg). Prior to IV infusion, ansuvimab must first be diluted in a 0.9% sodium chloride injection or 5% dextrose injection and then reconstituted with sterile water. Ansuvimab can be administered through a peripheral catheter or central line solution, but it should not be infused as an IV push or bolus (Jacob et al., 2020).

### 7.1 Pregnant women

EBOV infection is considered a life-threatening condition for pregnant women and affects both the mother and fetus. The majority of pregnancies with EBOV infection lead to adverse maternal/fetal outcomes, including premature birth, stillbirth, miscarriage, or neonatal death. Hence, pregnant women with EBOV infection should not postpone or refuse treatment (Ebanga, 2020). In the early phase of the PALM clinical study, the mortality rates of EVD were compared between subjects receiving ansuvimab, REGN-EB3, or remdesivir and patients who were treated with ZMapp. Pregnant women were also included in this study. The results revealed that both ansuvimab and REGN-EB3 significantly reduced mortality when compared to ZMapp; the efficacy of these treatments in pregnant women was not detected due to early termination of the trial (Mulangu et al., 2019). As neutralizing mAbs, such as ansuvimab, have the ability to transfer across the placenta, it might have the potential to pass from the mother to the growing fetus (Authors Anonymous, 2021).

### 7.2 Breastfeeding women

EBOV has been detected in blood and body fluids such as breast milk. Therefore, to reduce the risk of postnatal transmission of EBOV infection, the Centers for Disease Control and Prevention (CDC) has recommended mothers with a suspected or confirmed EBOV, or those who have recently recovered from EBOV infection, to avoid breastfeeding their newborns (National Library of Medicine, 2006). The administration of ansuvimab during breastfeeding and its impact on breastfed children or its presence in human milk is not well understood (Ebanga, 2020). Although maternal IgG is found in human milk, the presence of IgG1 mAbs, such as ansuvimab in breast milk, seems unlikely; if it is present, the absorption of these antibodies is impossible as it is probably eliminated in the infant’s gastrointestinal tract (National Library of Medicine, 2006; Medina-Rivera et al., 2021).

### 7.3 Pediatric population

The safety and efficacy of ansuvimab for the treatment of EBOV infection have been studied in pediatric patients from birth to less than 18 years old. Evidence obtained from the PALM study, which included 54 pediatric subjects (1 day–17 years old), affirmed the use of ansuvimab for this age group. In the PALM study, the safety of the drug and the 28-day mortality results for pediatric subjects who were treated with ansuvimab were similar to adults (Mulangu et al., 2019; Meyers and Shah, 2021).

### 7.4 Geriatric population

There is limited published information regarding the efficacy and the safety profile of ansuvimab in elderly patients 65 and older. Due to the small number of participants in the PALM study, it has not been determined whether the safety profile of ansuvimab is different in the geriatric population compared to younger patients (Ebanga, 2020).

## 8 Adverse effects of ansuvimab administration in clinical trials

As clinical trials are conducted under different conditions, adverse events found in clinical trial studies for a drug differ from those found in the real world among patients. The results of an open-label phase I study conducted on healthy adults receiving 5, 25, and 50 mg/kg of ansuvimab indicated no adverse effects or death. Only four patients showed mild side effects, including muscle or joint pain, discomfort, nausea, headache, and chills, 3 days after the ansuvimab injection. These results also suggest that ansuvimab is safe and tolerable in healthy adults (Gaudinski et al., 2019). In 2018–2019, during an EBOV outbreak in the Democratic Republic of the Congo, the PALM clinical trial evaluated ansuvimab safety and efficacy in 173 participants including 119 adults and 54 pediatric patients. Approximately 29% of the subjects showed hypersensitivity, including pre-specified infusion-related events, and in at least 10% of the patients who received ansuvimab, fever was recorded as the most common adverse event. Furthermore, some pre-specified symptoms that were monitored daily included diarrhea, pyrexia, abdominal pain, and vomiting, which were reported in ≥40% of subjects. Other common adverse events were tachycardia, fast breathing, chills, and hypotension. According to the FDA recommendation, this drug should not be prescribed in combination with a live virus vaccine against EBOV (Mulangu et al., 2019; Liu et al., 2022). Given the side effects reported so far regarding the administration of ansuvimab, signs of systemic hypersensitivity reactions should be immediately reported by patients, who should also be educated about hypersensitivity reactions including infusion-associated events during and after infusion (Authors Anonymous, 2021).

## 9 Outcomes of ansuvimab administration in experimental and clinical trials

The first *in vivo* studies were the quantification of the titer of ansuvimab in a live virus plaque-reduction neutralization assay in Vero E6 cells and an EBOV GP lentivirus infectivity assay using HEK293 cells. The results showed that ansuvimab can neutralize *Zaire* EBOV Mayinga at an EC_50_ value of 0.06–0.09 μg/mL. Moreover, in the presence of effector cells, ansuvimab-mediated ADCC was observed in EBOV GP-transduced and non-transduced HEK293T target cells at an mAb dose of 0.03 μg/mL. The results of *in vivo* and *in vitro* studies, as well as the outcomes of an investigation conducted on rhesus macaques, showed that treatment with a single IV dose of ansuvimab (50 mg/kg) 5 days after infection could inhibit EBOV-infected animals from death. The safety profiles of ansuvimab support its direct testing in humans infected by EBOV (Corti et al., 2016; Misasi et al., 2016; Misasi and Sullivan, 2021). In a phase I trial (NCT03478891) that began in 2018, Gaudinski et al. (2019) assessed the safety, tolerability, and pharmacokinetic profile of a single IV injection of different doses of ansuvimab (5, 25, and 50 mg/kg) in 18 individuals aged 18–60 years old. In the 24 week follow-up, participants reported mild systemic symptoms with no unsolicited adverse events. In addition, with a half-life of 24.2 days and linear pharmacokinetics, the administration of ansuvimab showed no signs of developing antidrug antibodies. Following possible immunogenicity and safety in the phase I study, a phase II/III clinical trial (NCT03719586) was conducted on 681 patients that were up to 99 years old. The patients were divided into four groups to evaluate the safety and effectiveness of four drugs (ZMapp, remdesivir, ansuvimab, and REGN-EB3). The outcomes demonstrated that the 28-day case fatality rate of patients who were given ansuvimab was 35.1% compared to the control (49.4%). Additionally, in subjects with a high primary viral load (initial EBOV nucleoprotein Ct ≤ 22), the ansuvimab arm had a lower incidence of death (69.9%) *versus* patients who received other treatments (84.5%) (Mulangu et al., 2019). The results of the phase II/III clinical trial suggest the effectiveness of ansuvimab in lowering case fatality rates in patients who received therapy immediately after experiencing symptoms and for those who had lower baseline levels of creatinine or alanine aminotransferase or had low virus loads (Levine, 2019).

## 10 Conclusion

Ansuvimab is currently recognized as the most effective approved treatment option for EVD. Its efficacy in clinical trials has been promising compared to control treatments. However, further investigations are needed to assess the effects of this new mAb in different patients identified in outbreaks. There are still many questions about the effectiveness and safety profile of ansuvimab in vulnerable populations such as pregnant women, children and infants, and the elderly. Thus, the administration of this effective drug in the real world and outside clinical trials could provide insights into many unanswered questions. An important issue regarding EBOV is its genetic changes and associated drug resistance. Since no resistance to ansuvimab has been currently reported, a detailed examination of circulating viruses in every outbreak and an assessment of genomic variations are beneficial to better understand the mechanisms of resistance and its management.

## References

[B1] Authors Anonymous (2021). Infusion-associated HRI. AHFS® first Release™. Am. J. Health Syst. Pharm. 78 (8), 649–651. 10.1093/ajhp/zxab092 33787830

[B2] Authors Anonymous (2019). New antibodies best ZMapp in Ebola trial. Nat. Biotechnol. 37 (10), 1105. 10.1038/s41587-019-0284-y 31578509

[B3] Cagigi A. Misasi J. Ploquin A. Stanley D. A. Ambrozak D. Tsybovsky Y. (2018). Vaccine generation of protective Ebola antibodies and identification of conserved B-cell signatures. J. Infect. Dis. 218 (5), S528–S536. 10.1093/infdis/jiy333 30010811 PMC6455927

[B4] Carette J. E. Raaben M. Wong A. C. Herbert A. S. Obernosterer G. Mulherkar N. (2011). Ebola virus entry requires the cholesterol transporter Niemann–Pick C1. Nat 477 (7364), 340–343. 10.1038/nature10348 PMC3175325 21866103

[B5] Centers for Disease Control and Prevention (2014). 2014-2016 Ebola outbreak in West Africa. Available at: https://www.cdc.gov/vhf/ebola/about.html (Accessed October 25, 2022).

[B6] Chakraborty C. (2021). Therapeutics development for Ebola virus disease: A recent scenario. Cur Opin. Pharmacol. 60, 208–215. 10.1016/j.coph.2021.07.020 34464933

[B7] Chughtai A. Barnes M. Macintyre C. (2016). Persistence of Ebola virus in various body fluids during convalescence: Evidence and implications for disease transmission and control. Epidemiol. Infect. 144 (8), 1652–1660. 10.1017/S0950268816000054 26808232 PMC4855994

[B8] Corti D. Misasi J. Mulangu S. Stanley D. A. Kanekiyo M. Wollen S. (2016). Protective monotherapy against lethal Ebola virus infection by a potently neutralizing antibody. Science 351 (6279), 1339–1342. 10.1126/science.aad5224 26917593

[B9] Crozier I. Britson K. A. Wolfe D. N. Klena J. D. Hensley L. E. Lee J. S. (2022). The evolution of medical countermeasures for Ebola virus disease: Lessons learned and next steps. Vaccines (Basel) 10 (8). 10.3390/vaccines10081213 PMC9415766 36016101

[B10] Di Paola N. Sanchez-Lockhart M. Zeng X. Kuhn J. H. Palacios G. (2020). Viral genomics in Ebola virus research. Nat. Rev. Microbiol. 18 (7), 365–378. 10.1038/s41579-020-0354-7 32367066 PMC7223634

[B11] Ebanga (2020). Prescribing information. Ridgeback Biotherapeutics, LP. Available at: https://www.accessdata.fda.gov/drugsatfda_docs/label/2020/761172s000lbl (Accessed October 25, 2022).

[B12] FDA (2020). FDA approves first treatment for Ebola virus. Available at: https://www.fdagov/news-events/press-announcements/fda-approves-first-treatment-Ebola-virus (Accessed October 25, 2022).

[B13] Feldmann H. Geisbert T. W. (2011). Ebola haemorrhagic fever. Lancet 377 (9768), 849–862. 10.1016/S0140-6736(10)60667-8 21084112 PMC3406178

[B14] Finch C. L. Dyall J. Xu S. Nelson E. A. Postnikova E. Liang J. Y. (2021). Formulation, stability, pharmacokinetic, and modeling studies for tests of synergistic combinations of orally available approved drugs against Ebola virus *in vivo* . Microorganism 9 (3), 566. 10.3390/microorganisms9030566 PMC7998926 33801811

[B15] Gaudinski M. R. Coates E. E. Novik L. Widge A. Houser K. V. Burch E. (2019). Safety, tolerability, pharmacokinetics, and immunogenicity of the therapeutic monoclonal antibody mAb114 targeting Ebola virus glycoprotein (VRC 608): An open-label phase 1 study. Lancet 393 (10174), 889–898. 10.1016/S0140-6736(19)30036-4 30686586 PMC6436835

[B16] Ghosh S. Saha A. Samanta S. Saha R. P. (2021). Genome structure and genetic diversity in the Ebola virus. Cur Opin. Pharmacol. 60, 83–90. 10.1016/j.coph.2021.06.010 34364102

[B17] Goldstein T. Anthony S. J. Gbakima A. Bird B. H. Bangura J. Tremeau-Bravard A. (2018). The discovery of Bombali virus adds further support for bats as hosts of ebolaviruses. Nat. Microbiol. 3 (10), 1084–1089. 10.1038/s41564-018-0227-2 30150734 PMC6557442

[B18] Jacob S. T. Crozier I. Fischer W. A. Hewlett A. Kraft C. S. Vega M-AdL. (2020). Ebola virus disease. Nat. Rev. Dis. Prim. 6 (1), 13–31. 10.1038/s41572-020-0147-3 32080199 PMC7223853

[B19] Kadanali A. Karagoz G. (2015). An overview of Ebola virus disease. North Clin. Istanb 2 (1), 81–86. 10.14744/nci.2015.97269 28058346 PMC5175058

[B20] Krishnan A. Miller E. H. Herbert A. S. Ng M. Ndungo E. Whelan S. P. (2012). Niemann-Pick C1 (NPC1)/NPC1-like1 chimeras define sequences critical for NPC1's function as a flovirus entry receptor. Viruses 4 (11), 2471–2484. 10.3390/v4112471 23202491 PMC3509659

[B21] Lee A. (2021). Ansuvimab: First approval. Drugs 81 (5), 595–598. 10.1007/s40265-021-01483-4 33751449 PMC7983082

[B22] Lee J. E. Fusco M. L. Hessell A. J. Oswald W. B. Burton D. R. Saphire E. O. (2008). Structure of the Ebola virus glycoprotein bound to an antibody from a human survivor. Nat 454 (7201), 177–182. 10.1038/nature07082 PMC2700032 18615077

[B23] Levine M. M. (2019). Monoclonal antibody therapy for Ebola virus disease. N. Engl. J. Med. 381, 2365–2366. 10.1056/NEJMe1915350 31774948

[B24] Liu C-H. Hu Y-T. Wong S. H. Lin L-T. (2022). Therapeutic strategies against Ebola virus infection. Viruses 14 (3), 579. 10.3390/v14030579 35336986 PMC8954160

[B25] Malvy D. McElroy A. K. de Clerck H. Günther S. van Griensven J. (2019). Ebola virus disease. Lancet 393 (10174), 936–948. 10.1016/S0140-6736(18)33132-5 30777297

[B26] Medina-Rivera M. Centeno‐Tablante E. Finkelstein J. L. Rayco‐Solon P. Peña‐Rosas J. P. Garcia‐Casal M. N. (2021). Presence of Ebola virus in breast milk and risk of mother‐to‐child transmission: Synthesis of evidence. Ann. N. Acad. Sci. 1488 (1), 33–43. 10.1111/nyas.14519 PMC8048832 33113592

[B27] Meyers R. Shah P. (2021). What’s new in children’s drugs. Contemp. Pediatr. 38 (12), 20–27.

[B28] Misasi J. Gilman M. S. Kanekiyo M. Gui M. Cagigi A. Mulangu S. (2016). Structural and molecular basis for Ebola virus neutralization by protective human antibodies. Science 351 (6279), 1343–1346. 10.1126/science.aad6117 26917592 PMC5241105

[B29] Misasi J. Sullivan N. J. (2021). Immunotherapeutic strategies to target vulnerabilities in the Ebolavirus glycoprotein. Immun 54 (3), 412–436. 10.1016/j.immuni.2021.01.015 33691133

[B30] Mulangu S. Dodd L. E. Davey R. T. Jr Tshiani Mbaya O. Proschan M. Mukadi D. (2019). A randomized, controlled trial of Ebola virus disease therapeutics. N. Engl. J. Med. 381 (24), 2293–2303. 10.1056/NEJMoa1910993 31774950 PMC10680050

[B31] Mulangu S. Mbala-Kingebeni P. Mbaya O. T. (2022). Antibody use during an outbreak of Ebola virus disease in the democratic republic of Congo, 2020. N. Engl. J. Med. 386 (12), 1188–1191. 10.1056/NEJMc2113505 35320651

[B32] National Library of Medicine (2006). Drugs and lactation database (LactMed). Bethesda (MD): National Library of Medicine. Ansuvimab. Available at: https://ncbi.nlm.nih.gov/books/NBK566931/ .

[B33] Paessler S. Walker D. H. (2013). Pathogenesis of the viral hemorrhagic fevers. Annu. Rev. Pathol. 8 (1), 411–440. 10.1146/annurev-pathol-020712-164041 23121052

[B34] Qiu X. Wong G. Audet J. Bello A. Fernando L. Alimonti J. B. (2014). Reversion of advanced Ebola virus disease in nonhuman primates with ZMapp. Nat 514 (7520), 47–53. 10.1038/nature13777 PMC4214273 25171469

[B35] Ridgeback Biotherapeutics (2021). EBANGA (ansuvimab-zykl): US prescribing information. Available at: https://dailymed.nlm.nih.gov/ (Accessed October 25, 2022).

[B36] DRUGBANK (2021). New eBook: Your guide to quality drug data. Available at: https://go.drugbank.com/drugs/DB16385 .

[B37] Sivanandy P. Jun P. H. Man L. W. Wei N. S. Mun N. F. K. Yii C. A. J. (2022). A systematic review of Ebola virus disease outbreaks and an analysis of the efficacy and safety of newer drugs approved for the treatment of Ebola virus disease by the US Food and Drug Administration from 2016 to 2020. J. Infect. Public Health 15, 285–292. 10.1016/j.jiph.2022.01.005 35085865

[B38] Traggiai E. Becker S. Subbarao K. Kolesnikova L. Uematsu Y. Gismondo M. R. (2004). An efficient method to make human monoclonal antibodies from memory B cells: Potent neutralization of SARS coronavirus. Nat. Med. 10 (8), 871–875. 10.1038/nm1080 15247913 PMC7095806

[B39] US Food and Drug Administration (2022). Drug trials snapshots: INMAZEB. Available at: https://www.fda.gov/drugs/drug-approvals-and-databases/drug-trialssnapshots-inmazeb (Accessed October 25, 2022).

[B40] Wang R. Zhang H. Peng C. Shi J. Zhang H. Gong R. (2021). Identification and characterization of a novel single domain antibody against Ebola virus. Virol. Sin. 36 (6), 1600–1610. 10.1007/s12250-021-00454-z 34632543 PMC8502631

[B41] Wilson J. A. Hevey M. Bakken R. Guest S. Bray M. Schmaljohn A. L. (2000). Epitopes involved in antibody-mediated protection from Ebola virus. Science 287 (5458), 1664–1666. 10.1126/science.287.5458.1664 10698744

[B42] World Health Organization (2020). Ebola virus disease. WHO. Available at: https://www.who.int/news-room/fact-sheets/detail/ebola-virus-disease (Accessed on December 28, 2020).

[B43] World Health Organization (2022). Ebola virus disease. Available at: https://www.who.int/health-topics/ebola/ (Accessed October 25, 2022).

